# Supplementary figures and images for: Mechanisms of generation of membrane potential resonance in a neuron with multiple resonant ionic currents
Source: PLoS Comput Biol. 2017 Jun 5;13(6):e1005565. doi: 10.1371/journal.pcbi.1005565 (PMC5476304; doi:10.1371/journal.pcbi.1005565)

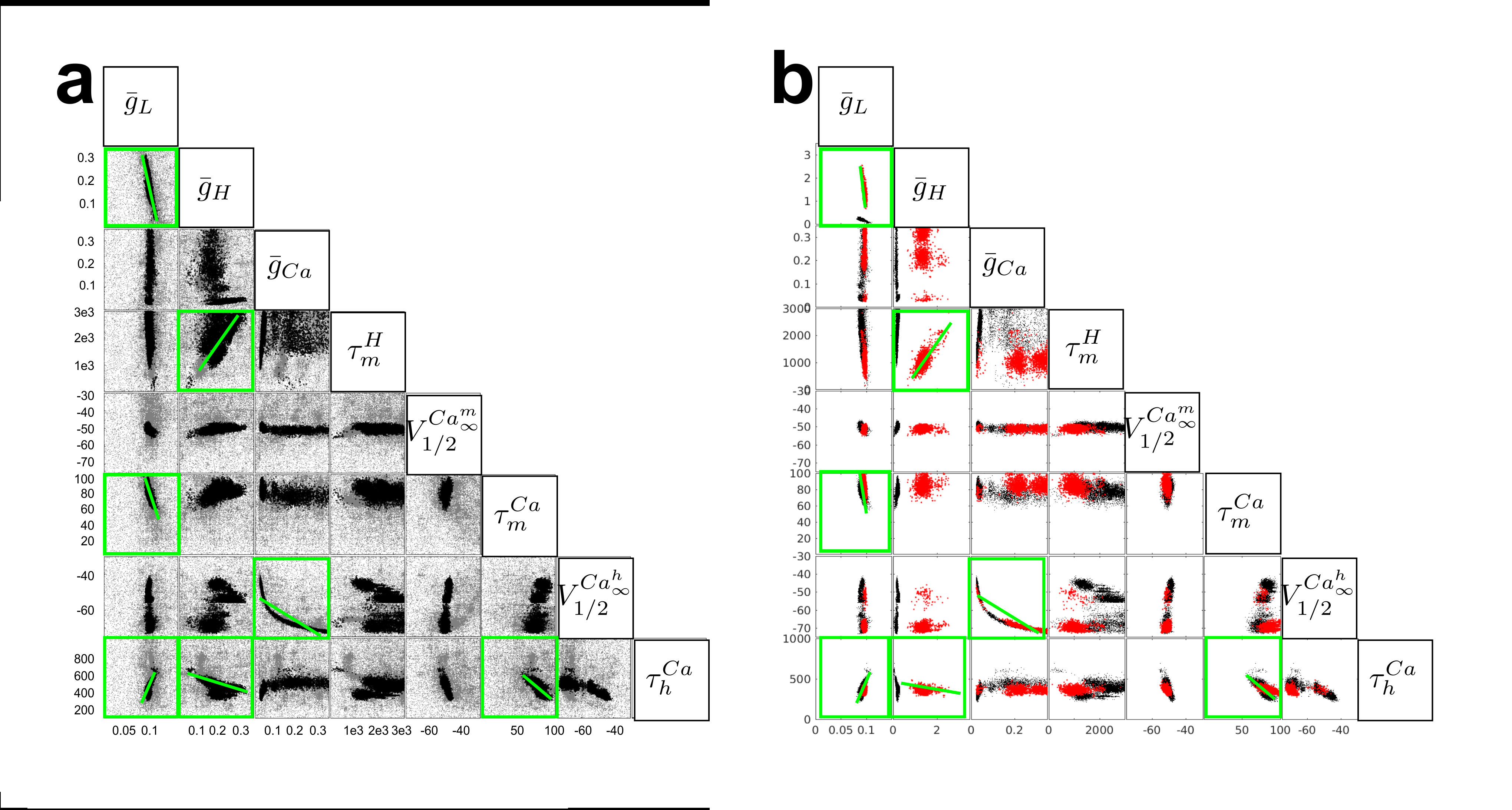

Supplement: S1 Fig — a. Correlations shown in Fig 8b with V1/2H∞m at -70 mV. b. Correlations obtained with V1/2H∞m set to -96 mV (red dots). MOEA was run only once in this case, compared to 5 times in panel a (hence the difference in the number of points). Black dots are the same as panel a. Note that the values of g¯H in this case are about 10 times larger than those in panel a, but the correlations (green boxes) remain intact. More importantly, the range of parameters other than g¯H is exactly the same in both cases. (TIF) [file pcbi.1005565.s001.tif]

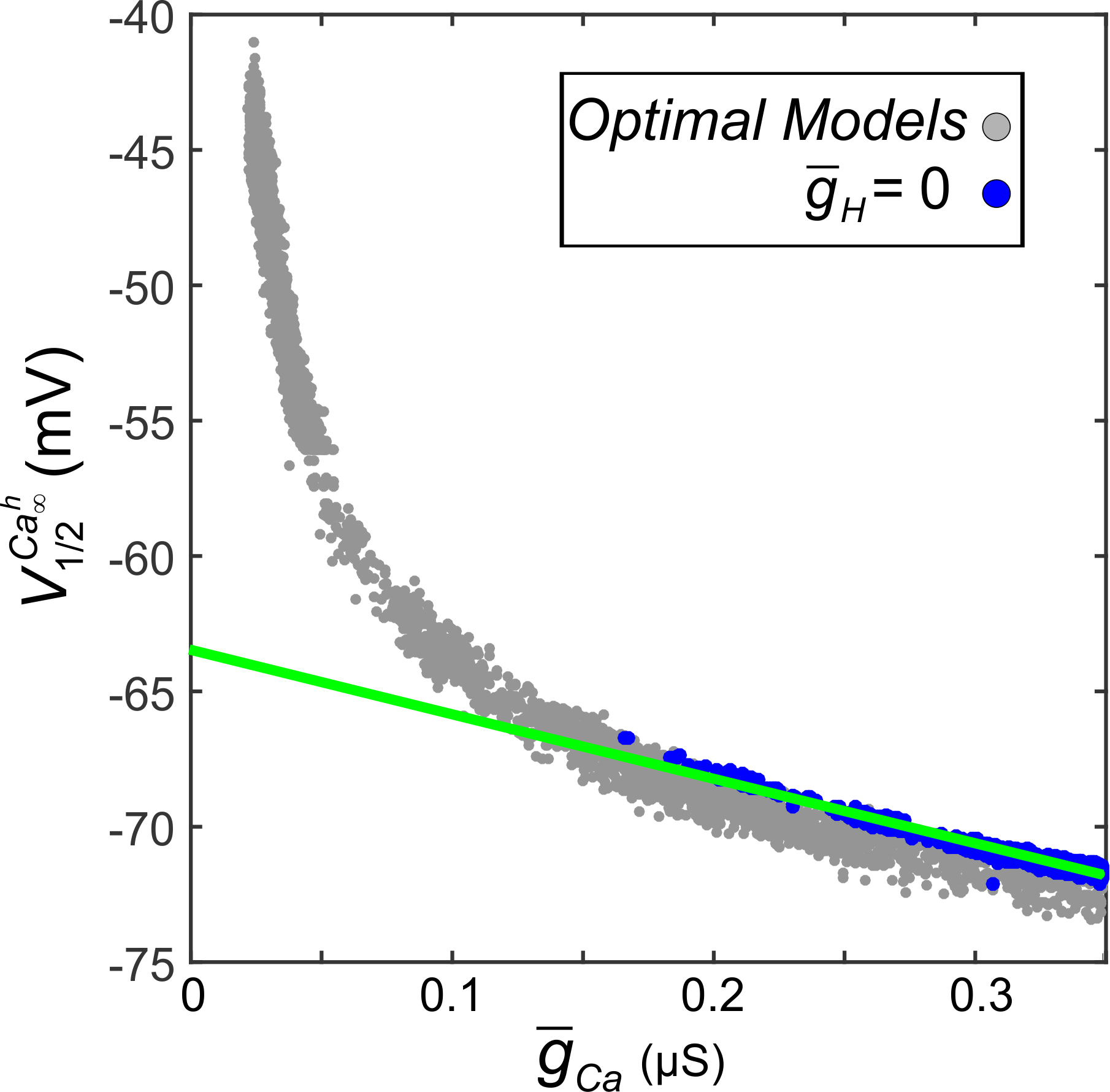

Supplement: S2 Fig — Parameter values for the optimal models in g¯Ca−V1/2Ca∞h space shown for all models (grey dots) and those without IH (blue dots). We removed IH by setting g¯H = 0, and ran the MOEA multiple times using the same Z- and φ-profiles to constrain the ICa parameters. A linear fit (green) shows that, when g¯H = 0, the relationship between g¯Ca−V1/2Ca∞h is linear and matches a narrow range of the high g¯Ca values in Fig 6c. (TIF) [file pcbi.1005565.s002.tif]
